# Role of Global Left Ventricle Longitudinal Strain and Cardiac Biomarkers in the Early Detection of Cancer Therapy-Related Dysfunction in Patients Treated With Cardiotoxic Chemotherapeutic Drugs in a Cardio-Oncology Clinic

**DOI:** 10.7759/cureus.71766

**Published:** 2024-10-18

**Authors:** Noha H Shaaban, Rasha M Abayazeed, Mohamed A Sobhy, Eman M Elsharkawy, Basma A Hammad

**Affiliations:** 1 Cardiology, Alexandria University, Alexandria, EGY; 2 Cardiology, Royal Brompton and Harefield Hospitals, London, GBR

**Keywords:** 2d gls, anthracyclines, cardioprotective treatments, ctrcd, nt pro bnp

## Abstract

Background

Breast cancer (BC) affects many women, and with the prevalence of anthracyclines (AC) used in treatment, cardiotoxicity is a commonly encountered problem.

Objective

The aim is to early detect subclinical cancer therapy-related cardiac dysfunction (CTRCD) using noninvasive imaging techniques and cardiac biomarkers.

Methods

Eighty-eight patients with cancer who planned to receive AC or trastuzumab (TZB) were enrolled. Baseline screening included two-dimensional (2D) transthoracic echocardiogram (TTE), global longitudinal strain (GLS), cardiac troponin I (cTnI), and N-terminal pro-brain natriuretic peptide (NT-ProBNP) measurements. Follow-up was done at three and six months to early detect CTRCD.

Results

Twenty-six patients developed CTRCD, defined by a relative decline in GLS and left ventricular ejection fraction (LVEF). The percentage of change in GLS from baseline and at three and six months was able to detect CTRCD in both groups in our population, which was >16.6% at three months with a p-value of <0.001* and CI 0.783-0.934 and >10.10% at six months with a p-value of <0.001* and CI 0.765-0.935. At three months, GLS values of ≤-18.6 were able to detect CTRCD with a p-value of <0.001* and CI 0.673 0.885. Compared to patients who did not develop CRTD, patients with mild asymptomatic CTRCD had double levels of NT-ProBNP with a median of (99.5) (interquartile range (IQR): 44.0-154.0) at three months.

Conclusion

The relative decline of GLS and elevation of NT-proBNP were able to diagnose patients with subclinical CTRCD in patients receiving AC with early start of cardioprotective treatments.

## Introduction

Breast cancer (BC) is the most common type of cancer in women and the first cause of cancer death among them. In Egypt, it constitutes 33% of female cancer cases, and more than 22,000 new cases are diagnosed each year [1]. Advances in cancer detection and treatment resulted in increased cancer survivors. Cancer therapy-related cardiac dysfunction (CTRCD) is nowadays a frequently encountered clinical presentation, and transthoracic echocardiography (TTE) is usually the routine imaging modality of its screening and detection [2,3]. The use of anthracyclines (AC) (doxorubicin, daunorubicin, and epirubicin) in the cancer treatment of patients with solid tumors have significantly improved their prognosis; however, they have irreversible dose-dependent cardiotoxic adverse effects [4-6]. Hence, withholding AC due to suspected toxicity could negatively impact the prognosis of those patients in low-income countries where there are unavailable, less toxic treatments.

The prototypical cardiac biomarkers of AC toxicity are cardiac troponins (cTn) and natriuretic peptides (NPs). Their rise can sometimes detect left ventricular dysfunction (LVD) earlier than echocardiography, and pretreatment levels can predict further CTRCD development [7,8]. On the other hand, human epidermal receptor 2 (HER 2) antagonists such as trastuzumab (TZB) are well known to cause reversible, not dose-dependent cardiotoxicity (type 2) [9], resulting in temporary LVD during treatment [6].

Multimodality cardiac imaging is the most widely used technique for monitoring patients and the diagnosis of CTRCD [3]. Given the challenges with serial left ventricular ejection fraction (LVEF) measurements, more sensitive methods to early detect and confirm cardiac dysfunction should be considered, including global longitudinal strain (GLS) and cardiac biomarkers (e.g., cTn and NPs) [3, 10].

Global longitudinal strain is now recommended as class I by the European Society of Cardiology (ESC) guidelines to be done at baseline and during the follow-up of patients on chemotherapy [11].

Similar to GLS, elevations in cTn and NP levels were proved to be useful for early detection of cardiotoxicity [12].

The purpose of our study was to evaluate the role of cardiac biomarkers and noninvasive imaging in the early detection of LVD in Egyptian patients receiving cardiotoxic drugs.

This article was previously posted to the Authorea preprint server on August 31, 2024.

## Materials and methods

This is a prospective study performed from January 2020 to June 2023 in the Alexandria University cardio-oncology clinic in Alexandria, Egypt. A detailed clinical assessment was done by a cardiologist and an oncologist [13].

All patients underwent two-dimensional (2D) TTE and 2D speckle tracking echocardiography (STE) before initiating AC-based treatment (T0), after three months (T1), and after six months (T2) of AC/TZB-based chemotherapy. All the images were obtained with an ultrasonography system iE 33 machine (Philips Medical Systems, Andover, MA, USA) and the EPIQ system (version CVx, Philips Medical Systems) using a fully sampled matrix transducer by one investigator, where the three intended studies were done on the same machine and processed on the same QLab versions 10 and 13 (Figure 53, LLC, Baltimore, MD, USA).

The 2D TTE was performed according to the current recommendations [14]. Assessment of left ventricular (LV) global and segmental longitudinal strain: after optimizing image quality, maximizing frame rate, and minimizing foreshortening [11,15-17].

In all regimens, doxorubicin was given via a 30-minute intravenous infusion. The used regimens included doxorubicin followed by cyclophosphamide, trastuzumab, Taxol, or hormonal treatment, except for two patients who received only trastuzumab treatment.

We defined CTRCD according to the latest ESC 2022 cardio-oncology guidelines [13]. Given that none of our patients had symptomatic dysfunction, we used the criteria of asymptomatic dysfunction to categorize the patients who developed asymptomatic dysfunction during their follow-up into a) mild dysfunction: LVEF ≥50% and a new relative decline in GLS by >15% from baseline and/or new rise in cardiac biomarkers, b) moderate dysfunction: new LVEF reduction by ≥10 percentage points to an LVEF of 40-49% or new LVEF reduction by <10% points to a value 0f 40-49% and either new relative decline in GLS by >15% from baseline or new rise in cardiac biomarkers, and c) severe dysfunction: new LVEF reduction to <40%.

Patients diagnosed with subclinical CTRCD were prescribed cardioprotective treatment in the form of angiotensin-converting enzyme (ACE) inhibitors (ramipril) and beta blockers (bisoprolol), starting with the lowest tolerated dose and increasing according to the clinical assessment during the follow-up visits [13].

Study patients’ blood samples (three milliliters of blood) were taken before chemotherapy (T0), at three months (T1), and at six months (T2) during the scheduled follow-ups. Samples were sent to a special laboratory outside the University of Alexandria.

Tests were performed with standard clinical practice methodology. The samples were used to measure serum N-terminal pro-brain natriuretic peptide (NT-ProBNP) and cardiac troponin I (cTnI) [18].

Statistical analysis

Data were analyzed using IBM SPSS Statistics software, version 20.0. (IBM Corp., Armonk, NY) Qualitative data were described using numbers and percentages. The Kolmogorov-Smirnov and Shapiro-Wilk tests were used to verify the normality of the distribution. Quantitative data were described using range (minimum and maximum), mean, standard deviation, median, and interquartile range (IQR). Chi-square test for categorical variables to compare between different groups Monte Carlo correction for chi-square when more than 20% of the cells have an expected count less than five. One-way ANOVA test for normally distributed quantitative variables to compare between more than two groups, and post hoc test (Tukey) for pairwise comparisons. Kruskal-Wallis test for not normally distributed quantitative variables to compare between more than two studied groups, and post hoc (Dunn's multiple comparisons test) for pairwise comparisons. The significance of the obtained results was judged at the 5% level.

Ethical approval and informed consent

The study was done according to the Declaration of Helsinki, and ethical approval was granted by the Ethics Committee of the Faculty of Medicine, Alexandria University, Alexandria, Egypt, with the institutional review board (IRB) number: 00012098. Written informed consent was obtained from all participants.

Inclusion and exclusion criteria

Table 1 shows the inclusion and exclusion criteria of the study.

**Table 1:** Inclusion and exclusion criteria of the study AC: anthracyclines; CMRI: cardiac magnetic resonance imaging; TZB: trastuzumab; LVEF: left ventricular ejection fraction

Inclusion criteria	Exclusion criteria
Informed consent before any study-mandated procedure	Preexisting left ventricular dysfunction defined as LVEF <50% at baseline study.
Patients age >18 years	Valvular disease (stenotic and regurgitant lesions) grade II or more
Recent cancer diagnosis	Cardiovascular event or revascularization within 12 weeks
Receiving chemotherapy (AC or TZB)	Systemic inflammatory disease (rheumatoid arthritis, systemic lupus erythematosus, systemic sclerosis).
No existing left ventricular dysfunction at baseline	Contraindications to CMRI
	Cancer survivors who previously received chemotherapeutic drugs or radiotherapy
	Prior history of chest irradiation
	Chemotherapeutic agents other than (AC or TZB)
	Atrial fibrillation, multiple premature ventricular or atrial contractions, or any other condition that would interfere with strain imaging
	Left bundle branch block

## Results

All patients underwent clinical evaluation, serum biomarkers measurement, as well as 2D TTE and GLS by STE at baseline (T0), after three months (T1), and after six months (T2) of starting chemotherapy. Table 2 presents the baseline demographics.

**Table 2:** Demographic data of the study population This table demonstrates the baseline demographics, risk factors, and clinical parameters among the study population. BC: breast cancer; BSA: body surface area; DM: diabetes mellitus; HR: heart rate; HTN: hypertension, No.: number, Sig: significance; ^MC^p: Monte Carlo p-values

Demographic data	Total sample (n= 88)	No dysfunction (n = 62)	Asymptomatic dysfunction		Test of sig.	p-value
			Mild (n = 18)	Moderate (n = 8)		
		No. (%)	No. (%)	No. (%)		
Gender						
Male	7 (8%)	4 (6.5%)	1(5.6%)	2 (25%)	χ^2^=3.167	^MC^p=0.185
Female	81 (92%)	58 (93.5%)	17 (94.4%)	6 (75%)		
Age (years)	43.33 ± 12.36	42.65 ± 11.41	47.06 ± 14.85	40.25 ± 13.37	F=1.165	0.317
Type of malignancy						
Other types	15 (17%)	10 (16.1%)	3 (16.7%)	2 (25%)		^MC^p=0.724
BC	73 (83%)	52 (83.9%)	15 (83.3%)	6 (75%)		
Baseline HR	81.95 ± 15.40	83.10 ± 14.25	76.0 ± 17.43	86.50 ± 17.68	F=1.904	0.155
Baseline risk factors						
DM	3 (3.4%)	1 (1.6%)	1 (5.6%)	1 (12.5%)	χ^2^=3.549	^MC^p=0.123
HTN	14 (15.9%)	9 (14.5%)	3 (16.7%)	2 (25%)	χ^2^=0.988	^MC^p=0.718
Smoking	0 (0%)	0 (0%)	0 (0%)	0 (0%)	–	–
Family history	1 (1.1%)	0 (0%)	0 (0%)	1 (12.5%)	χ^2^=5.478	^MC^p=0.095
BSA	1.89 ± 0.24	1.89 ± 0.25	1.88 ± 0.21	1.90 ± 0.24	F=0.010	0.990

A total of 26 patients (30%) met the criteria of CTRCD; this represents quite a large number of patients receiving chemotherapy (Table 2). The study population was divided into three groups according to the occurrence of CTRCD: Group 1: no dysfunction (n = 62); Group 2: mild asymptomatic dysfunction (n = 18); Group 3: moderate asymptomatic dysfunction (n = 8).

We found that patients who developed mild asymptomatic dysfunction had a higher level of NT-ProBNP at the three-month follow-up with a median of 99.5 with a p-value of 0.037; this value was double their baseline value, and it normalized during the six-month follow-up (Table 3). Regarding the echocardiographic findings, at three months, indexed LV end-systolic volumes (LVESVi) were significantly increased only in patients who developed moderate asymptomatic dysfunction with a p-value of 0.015, moreover at six months both groups with mild & moderate asymptomatic dysfunction showed significantly increased LVESVi with a p-value of 0.019.

**Table 3:** Cardiac biomarkers among the study population This table shows the values of cardiac troponin I and NT-ProBNP among the study population Sig.: significance; *: statistically significant; p: p-value for comparing the three studied groups; p_1_: p-value for comparing between no dysfunction and mild asymptomatic dysfunction; p_2_: p-value for comparing between no dysfunction and moderate asymptomatic dysfunction; p_3_: p-value for comparing between mild and moderate asymptomatic dysfunction

Biomarkers	Total sample	No dysfunction	Asymptomatic dysfunction		Test of sig.	p-value
			Mild	Moderate		
Troponin						
Baseline	0.11 (0.10 – 0.12)	0.11 (0.10 – 0.12)	0.11 (0.10 – 0.12)	0.10 (0.10 – 0.13)	H = 0.552	0.759
3 months	0.11 (0.10 – 0.12)	0.11 (0.10 – 0.12)	0.11 (0.10 – 0.11)	0.10 (0.10 – 0.11)	H =0.668	0.716
6 months	0.11 (0.10 – 0.12)	0.11 (0.10 – 0.11)	0.10 (0.10 – 0.13)	0.10 (0.10 – 0.12)	H =0.192	0.909
NT-ProBNP						
Baseline	58.0 (34.0 – 80.0)	52.50 (34.0 – 68.0)	80.10 (62.0 – 105.0)	57.95 (20.0 – 128.5)	H =5.467	0.065
3 months	51.30 (30.75 – 87.25)	50.20 (30.10 – 83.0)	99.50 (44.0 – 154.0)	39.0 (26.25 – 87.0)	H =6.613^*^	0.037^*^
Significance between the groups		p_1_=0.015^*^, p_2_=0.643,p_3_=0.530				
6 months	53.0 (30.05 – 83.0)	45 (28.0 – 77.80)	72 (54.50 – 115.0)	52 (23.15 – 58.50)	H= 5.045	0.080

Regarding the STE, the mean GLS was -21.19 ± 2.31 at baseline among the three studied groups; interestingly, the mean GLS was significantly lower in patients with moderate asymptomatic dysfunction (group three) with a mean value of 20.15 ± 2.21 p-value of 0.005. This group later developed CTRCD (Table 4). At the three-month follow-up (T1), there was a statistically significant lower GLS value among Groups 2 and 3 with a p-value <0.001. The mean GLS value among Group 1 was 20.62 ± 2.56, Group 2 was -18.84 ± 1.84, and Group 3 was 16.25 ± 1.99. At the six-month follow-up (T2), there were still statistically significant lower GLS values in Groups 2 and 3, where the mean GLS among Groups 1, 2, and 3 were -21.05 ± 2.53, -18.77 ± 2.73, and 18.83 ± 2.53, respectively, with a p-value of 0.005. There was no significant difference between the indexed cumulative doses among the three groups. Eighty-two patients received AC before their first follow-up; the remaining six patients who did not receive AC at three months had received TZB (four patients), Taxol, and carboplatin (two patients), followed by AC (Tables 3, 4).

**Table 4:** Comparison between the three studied groups according to LVEF, indexed volumes, and GLS This table shows the values of LVEF, GLS, LVEDVi, and LVESVi among the study population. GLS: global longitudinal strain; LVEF: left ventricular ejection fraction; LVEDVi: left ventricular end-diastolic volume indexed; LVESVi: left ventricular end-systolic volume indexed *: statistically significant; p: p-value for comparing between the three studied groups; p_1_: p-value for comparing between no dysfunction and mild asymptomatic dysfunction; p_2_: p-value for comparing between no dysfunction and moderate asymptomatic dysfunction; p_3_: p-value for comparing between mild and moderate asymptomatic dysfunction

	Biomarkers	Total sample	No dysfunction	Asymptomatic dysfunction		F	p
				Mild	Moderate		
LVEF	Baseline	61.15 ± 5.85	60.84 ± 5.74	62.94 ± 5.69	59.56 ± 6.95	1.235	0.296
	3 months	58.65 ± 6.47	60.15 ± 5.65	58.09 ± 5.74	48.35 ± 4.47	15.944^*^	<0.001^*^
	Significance between groups		p_1_=0.357, p_2_<0.001^*^, p_3_<0.001^*^				
	6 months	58.92 ± 6.66	60.33 ± 6.31	57.80 ± 5.10	50.86 ± 6.62	7.708^*^	0.001^*^
	Significance between groups		p_1_=0.342, p_2_=0.001^*^, p_3_=0.041^*^				
LVEDVi	Baseline	49.07 ± 12.59	48.65 ± 12.81	48.42 ± 11.30	53.82 ± 14.21	0.623	0.539
	3 months	49.54 ± 11.46	49.20 ± 11.75	49.99 ± 11.88	51.15 ± 9.06	0.118	0.889
	6 months	52.67 ± 12.15	51.85 ± 12.15	54.20 ± 13.38	55.52 ± 10.18	0.423	0.657
LVESVi	Baseline	20.04 ± 6.15	20.14 ± 6.29	18.49 ± 6.05	22.71 ± 4.74	1.338	0.268
	3 months	21.09 ± 6.36	20.53 ± 6.27	20.30 ± 5.70	27.24 ± 5.82	4.448^*^	0.015^*^
	Significance between groups	p_1_=0.989, p_2_=0.012^*^,p_3_=0.024^*^					
	6 months	22.09 ± 6.21	21.38 ± 5.71	21.69 ± 5.94	28.28 ± 7.76	4.194^*^	0.019^*^
	Significance between groups		p_1_=0.791, p_2_=0.738, p_3_=0.970				
GLS	Baseline	21.19 ± 2.31	20.77 ± 2.11	23.06 ± 2.15	20.15 ± 2.21	9.148^*^	<0.001^*^
	Significance between groups		p_1_<0.001^*^, p_2_=0.714, p_3_=0.005^*^				
	3 months	19.85 ± 2.72	20.62 ± 2.56	18.84 ± 1.84	16.25 ± 1.99	13.863^*^	<0.001^*^
	Significance between groups		p_1_=0.018^*^, p_2_<0.001^*^,p_3_=0.033^*^				
	6 months	20.39 ± 2.74	21.05 ± 2.53	18.77 ± 2.73	18.83 ± 2.53	5.751^*^	0.005^*^
	Significance between groups		p_1_=0.010^*^, p_2_=0.121, p_3_=0.998				

Cardioprotective treatment was given to a total of 24 patients in Groups 2 and 3. After three months of cardioprotective treatment, 10 patients out of the two groups showed improvement in their LV systolic function >50% or improved GLS despite continuing their chemotherapeutic treatment regimens.

By using receiver operating characteristic (ROC) analysis, the most significant parameter to diagnose CTRCD in patients receiving AC was the percentage of change in GLS with a p-value <0.001. Moreover, absolute GLS values less than -18.6 at three months were found to be diagnostic of CTRCD with a sensitivity (Se), specificity (Sp), and positive and negative predictor values of 69.23, 80.33, 60, and 86, respectively, with a confidence interval of 0.673-0.885 and a p-value of <0.001*. However, at six months, the percentage change in GLS to diagnose CTRCD was 10.10%. The lower cutoff value derived at six months can be attributed to lower GLS at three months and a smaller number of patients developing CTRCD at six months.

## Discussion

This study showed an early incidence of CTRCD in cancer patients treated with AC-based chemotherapy. Our clinic is the first established multidisciplinary cardio-oncology clinic in Egypt, and its implementation saved critical cancer patients from the hazards of unrecognized cardiotoxicity of AC and TZB. In all the patients who had CTRCD, AC was the cornerstone of their treatment. The detection of early cardiotoxicity in this study was based on echocardiography during the treatment with AC/TZB chemotherapy.

In the present study, 18 out of the 88 patients (20%) who received AC-based chemotherapy exhibited mild asymptomatic LV dysfunction, and eight patients (9%) had moderate asymptomatic dysfunction.

The main findings of our study were: (1) patients with low normal values of baseline GLS are more prone to CTRCD even with normal LVEF; (2) early subclinical CTRCD is common in patients receiving AC-based treatment; (3) during AC-based chemotherapy, the increase in serum NT-ProBNP levels was more pronounced in patients who developed cardiotoxicity and were indicative of early LV dysfunction; and (4) starting cardioprotective treatment early in patients with CTRCD could prevent worsening of LV systolic function.

Anthracycline is a class of chemotherapies that are widely used. The risk of developing heart failure is increased with exposure to higher cumulative doses of AC, and it appears to be permanent due to apoptosis of myocytes, which lack the regenerative capacity (type 1 cardiotoxicity). However, recent research showed that AC can have early acute cardiotoxicity, which is reversible [19,20]. Anthracycline cardiotoxicity is associated with direct oxidative damage, and recently, topoisomerase II beta has been proposed as another key mediator [4,21-23]. In our study, we found that, though the median cumulative doxorubicin dose was 432 mg, quite low, early subclinical LV systolic dysfunction was still observed in 30% of the patients in the study population. For years, the LVEF has been used before and during cardiotoxic treatment. However, the decline of LVEF represents the late stages of CTRCD. Hence, thoughts were directed to early markers of subclinical dysfunction as measurement of peak GLS by STE and cardiac biomarkers [18].

In our study, we used standard 2D echocardiography to assess the LV systolic function among the patients before starting AC/TZ, which helped the diagnosis of early CTRCD by comparing the follow-up measurements to the baseline values. Changes in LVEF measured in points together with changes in GLS were compared to baseline values, which helped in classifying the patients into three groups. Group 1, where no dysfunction occurred guided by 2D LVEF and GLS; Group 2, where patients had LVEF >50% with GLS decline by >15%; and lastly, Group 3, where patients had lowered LVEF between 40% and 49%. In both groups two and three. Cardioprotective therapy in the form of bisoprolol and ramipril was initiated and continued during the period of chemotherapy. In our study, 18 patients showed a>15% decline in their GLS without a drop of LVEF below 50%. Interestingly, this subset of patients had doubled their baseline values of NT-ProBNP. Eight patients had lowered LVEF between 40%-49% (Group 3), and those patients continued their chemotherapy uninterrupted. During the follow-up, 10 patients showed improvement in their LVEF/GLS. Regarding STE parameters, a 16.6% and 10% change in GLS at three and six months of follow-up, respectively, was the cut-off limit to diagnose CTRCD. Also, absolute values of GLS ≤ -18.6 at three months were diagnostic of CTRCD. This relatively lower threshold might be explained by the low prevalence of CV risk factors in a predominantly female cohort and also the exclusion of patients with low baseline GLS.

By comparing our study to others in the literature, in a study by Raj et al. (2014), decreased baseline GLS<-18 was shown to be a predictor of CTRCD defined by more than 10 percentage points drop from the baseline LVEF [24]. Another prospective study in adults with lymphoma or leukemia demonstrated that GLS <-17.45% at a cumulative AC dose of >150 mg/m2 is an independent predictor of future CTRCD, defined as a decrease in the LVEF of >10% points to a value e <53% [25].

This finding is supported by the study of Ali et al. (2016), where a GLS threshold of −17.5% before AC therapy would have correctly identified 86% of the patients who would develop a cardiac event after the start of chemotherapy, defined as symptomatic heart failure or cardiac death [26].

In all the previously mentioned studies, the main focus of the research was baseline GLS and its validity in predicting cardiotoxicity; however, regarding monitoring GLS during cardiotoxic treatment, the Strain Surveillance of Chemotherapy for Improving Cardiovascular Outcomes (SUCCOUR) study remains a landmark trial to monitor cardiotoxicity by LVEF versus the percentage of change in GLS. It showed that there was a higher rate of cardioprotective treatment implementation using STE than standard 2D echocardiography; however, there was no statistical significance between groups regarding the occurrence of CTRCD [13].

In the last decade, landmark reports demonstrated the additive value of strain assessment after cardiotoxic treatments [19,27,28]. These results revealed that early decreases in deformation parameters during or after cancer therapy in adults allowed for to prediction of the subsequent LVEF deterioration. In a study by Sawaya et al. (2012), the authors found that patients with BC treated with AC, taxanes and TZB, GLS, and ultrasensitive troponin I measured at the completion of AC therapy were useful in the prediction of subsequent cardiotoxicity and may have helped guide treatment to avoid cardiac side-effects; patients who had dysfunction showed GLS <-19 after their AC doses [19].

The combination of GLS and cardiac biomarkers is believed to enhance the diagnosis of early cardiotoxicity. It is recommended to measure cardiac serum biomarkers, such as cTnI or cTnT and BNP/NT-ProBNP, during cancer therapies, including AC and others [3, 29]. Elevated NPs can detect early doxorubicin-induced cardiotoxicity because their higher levels can detect LV dysfunction earlier than LVEF by echocardiography [8]. In a study by Feola et al. (2011), patients who had a drop in LVEF of >10% during follow-up had a higher baseline BNP of 55.5 _ 72.3 pg/mL, and those with normal LVEF had a baseline of 26.1 _ 21.4 pg/mL (p = 0.07 hazard ratio (HR) 0.96-1) [7]. On the contrary, other studies that investigated the association between cardiotoxicity and BNP/NT-ProBNP showed different results [30]. However, going in line with our study, another Egyptian study done by Sulaiman et al. (2021) highlighted the relative reduction of LV-GLS combined with the relative elevation of NT-ProBNP was successful in defining subclinical subtle chemotherapy-induced cardiotoxicity after six weeks of the first chemotherapeutic agent administration, where they concluded that a 2.2 relative elevation of the NT-ProBNP was able to define a relative reduction of LV-GLS >15% by a 100% sensitivity and 81.8% specificity [21].

In our study, we initiated cardioprotective treatment in the form of bisoprolol and ramipril in patients fulfilling the criteria of CTRCD, and we observed that none of the patients who had mild or moderate degrees of CTRCD progressed to worse grades. Unfortunately, the relatively small number of those patients hinders the strong conclusion. The issue of starting cardioprotective treatment in patients on chemotherapy has been debatable for a very long time due to the heterogenicity of the results and the lack of large-scale randomized trials. In 2006, Cardinale et al. (2006) studied a group of cancer patients where the patients with elevated troponin levels received enalapril and the control group did not. The results were impressive; none of the patients in the enalapril group had cardiotoxicity; however, 43% of the control group had cardiotoxicity defined by >10% points drop in absolute LVEF reaching <50% [22]. Another aspect of the SUCCOUR trial is that it tests the implementation of cardioprotective treatment based not only on LVEF but also on GLS change [15]. There was no significant between-group difference observed at one year for the primary outcome (LVEF change) or in secondary outcomes such as change in GLS, even though the use of cardioprotective treatment was significantly higher in the GLS-guided arm. Another secondary endpoint, the incidence of CTRCD, was lower in the GLS-guided arm than in the standard-of-care arm. Given the lack of a control group not taking cardioprotective treatment, the results will remain speculative. Moreover, the cut-off points for diagnosing CTRCD were lower than those published in the ESC guidelines of cardio-oncology.

Cardiotoxicity is considered the most dangerous adverse reaction of doxorubicin-based chemotherapy in patients with cancer. Our study results show that AC-based chemotherapy not only causes chronic cardiac dysfunction but also can cause acute cardiotoxicity that, when early detected and treated, may be reversible [23,24].

Study limitations

One limitation of our study is that it is a relatively small sample and no control group assessed by only LVEF. Another limitation of our study is that the chemotherapy regimens were not homogenous among the groups. Also, troponin levels were not uniformly measured at a specific time schedule related to AC dose. Last but not least, the limited duration of our study may have contributed to the loss of more patients developing CTRCD after six months.

## Conclusions

Our study demonstrated that LV GLS may be used as an early diagnostic marker of anthracycline-induced subclinical LV cardiotoxicity together with increased levels of NT-ProBNP, enabling early start of CPT in those patients, which prevented further worsening of LVEF without interrupting the cancer treatment. Interestingly, some patients exhibited improvement in their LVEF and GLS.
